# Folate, Vitamin B_12_, and Homocysteine as Risk Factors for Cognitive Decline in the Elderly

**DOI:** 10.4306/pi.2008.5.1.36

**Published:** 2008-03-31

**Authors:** Jae-Min Kim, Sung-Wan Kim, Il-Seon Shin, Su-Jin Yang, Woo-Young Park, Sung-Jin Kim, Hee-Young Shin, Jin-Sang Yoon

**Affiliations:** 1 Department of Psychiatry, Chonnam National University Medical School, Gwangju, Korea.; 2 Clinical Trial Center, Chonnam National University Hospital, Gwangju, Korea.

**Keywords:** Folate, Vitamin B_12_, Homocysteine, Cognitive decline, Dementia

## Abstract

**Objective:**

Cross-sectional studies have shown that the dysregulation of one-carbon metabolism is associated with cognitive impairment. However, the findings of longitudinal studies investigating this association have been inconsistent. This study investigated the prospective associations between cognitive decline and the levels of folate, vitamin B_12_ and homocysteine both at baseline and over course of the study period.

**Methods:**

A total of 607 (83%) elderly individuals were selected from a group of 732 elderly individuals without dementia at baseline and followed over a 2.4-year study period. The Mini-Mental State Examination (MMSE) was administered to the subjects, and the serum levels of folate, vitamin B_12_ and homocysteine were assayed both at baseline and at follow-up examinations. Covariates included demographic data, disability, depression, alcohol consumption, physical activity, vascular risk factors, serum creatinine level, vitamin intake, and apolipoprotein E genotype.

**Results:**

Cognitive decline was associated with decreasing quintiles of folate at baseline, a relative decline in folate and an increase in homocysteine across the two examinations after adjustment for relevant covariates.

**Conclusion:**

These results suggest that folate and homocysteine are involved in the etiology of cognitive decline in the elderly.

## Introduction

Folate, vitamin B_12_, and homocysteine are involved in the one-carbon transfer (methylation) reactions that are necessary for the production of monoamine neurotransmitters, phospholipids, and nucleotides. Homocysteine may also have a direct neurotoxic effect. Therefore, hyperhomocysteinemia and deficiencies in folate and vitamin B_12_ may contribute to the pathogenesis of cognitive impairment. Several cross-sectional studies have found significant associations between the adverse profiles of these factors and cognitive impairment.^1^-^5^ However, the direction of cause and effect in these associations cannot be ascertained from these findings because changes in appetite and diet associated with cognitive impairment may result in nutritional deficiencies. The results from prospective studies have been controversial: some studies found significant associations between these factors and cognitive decline,^6^ while others did not.^7^ There has been little research into the changes in the factors associated with cognitive decline.

In a previous community study, we found that cognitive impairment was significantly associated with lower folate and higher homocysteine levels, but not with the levels of vitamin B_12_.^8^ We subsequently followed the study cohort over a 2-year period and investigated the associations between cognitive decline and the levels of folate, vitamin B_12_ and homocysteine at baseline and throughout the follow-up period.

## Methods

A community based prospective survey was carried out in Gwangju, South Korea between 2001 and 2003 in collaboration with the 10/66 Dementia in Developing Countries Research Program.^9^ All participants gave written formal informed consent at each examination. This study was approved by the Chonnam National University Hospital Institutional Review Board.

### Baseline evaluation

The sampling procedure and measurements of the baseline cross-sectional survey have been described previously.^10^,^11^ In brief, 732 community residents aged 65 or over within two geographic catchment areas (one urban, one rural) of Gwangju, South Korea were identified from national resident registration lists. The examinations included cognitive function assessments; blood samples for analyses of folate, vitamin B_12_, and homocysteine; and a formal assessment of potential confounding factors.

Cognitive functioning was assessed using the Korean version of the Mini-Mental State Examination (MMSE-K).^12^ The MMSE-K has been specifically developed and evaluated for the assessment of cognitive functioning in older Korean populations, with revised items taking low education levels and high rates of illiteracy into account.

Blood samples were collected in a fasting state and were obtained in the morning when possible. The blood samples were drawn into ethylenediaminetetra acetic acid (EDTA) tubes, centrifuged, separated into plasma aliquots, and stored at -70℃ within 2 hours of collection. Biochemical assays were carried out after three years. The levels of serum folate and vitamin B_12_ were determined using an immunoassay, and highperformance liquid chromatography was used to measure total plasma homocysteine levels. Apolipoprotein E (APOE) genotyping was carried out, and the samples were classified according to the presence or absence of the APOE e4 allele.

Demographic data on age, gender, and education level were recorded. Disability was assessed by the Korean version of the World Health Organization Disability Assessment Schedule II (WHODAS II).^13^ Depression was assessed using the community version of the Geriatric Mental State schedule (GMS).^14^,^15^ As in other studies, a 'stage one' (non-hierarchical) confidence level of 3 or above from the Automated Geriatric Examination for Computer Assisted Taxonomy (AGECAT) algorithm was used to define depression. Information on the subjects' smoking history and current smoking status was obtained. A lifetime history of alcohol consumption was obtained from the participants, and the information provided was confirmed by their family members if possible. A high intake of alcohol was defined on the basis of consumption over the previous three months of greater than 14 drinks per week for men or greater than 7 drinks per week for women. Information on daily physical activity, including both work and leisure activities, was obtained, and the subjects were classified as having a low level of physical activity on the basis of a predominantly sedentary lifestyle. For the assessment of vascular risk factors and disorders, a summary vascular risk score was calculated by summing self-reported disorders (stroke, heart disease, hypertension, diabetes), measured obesity (body mass index > 25 kg/m^2^) and hypercholesterolemia (fasting cholesterol > 200 mg/dl). Serum creatinine levels were also evaluated because impaired renal function may elevate serum metabolite levels independent of vitamin intake.

### Follow-up evaluation

The follow-up evaluation was carried out in 2003.^16^ The mean (SD) follow-up period was 2.4 (0.3) years. Attempts were made to follow-up all previous participants. The procedures used in the follow-up evaluation were identical to those used in the baseline assessment. The MMSE-K scores were reassessed, and changes in the MMSE-K scores (score in 2003 minus that in 2001) were calculated. Further blood samples for folate, vitamin B_12_ and homocysteine were collected, centrifuged within one hour and stored at -70℃. Assays were carried out after one year. Data on the use of vitamin supplements was obtained by taking an inventory of all prescription and nonprescription medications taken within the past one month.

### Statistical analysis

Associations between cognitive decline and baseline characteristics were investigated using t-, Pearson's correlation, or Spearman's correlation tests as appropriate. Univariate associations between changes in the MMSE score (as a continuous dependent variable) and baseline quintiles of folate, vitamin B_12_ and homocysteine were estimated by analysis of variance (ANOVA). To investigate the associations between changes in the levels of folate, vitamin B_12_ and homocysteine, the changes in these three values (value in 2003 minus that in 2001) were also recategorized by quintiles. Univariate associations between changes in the MMSE score and changes in the quintiles of folate, vitamin B_12_ and homocysteine were also estimated by ANOVA. For the multivariate analysis, B coefficients (with 95% CI) were calculated for the associations between changes in the MMSE score from baseline and changes in the quintiles of folate, vitamin B_12_ and homocysteine in linear regression models after adjustment for relevant independent variables. Statistical analyses were carried out using SPSS 12.0 software.

## Results

A total of 607 (83%) of the 732 participants recruited at baseline completed all follow-up evaluations and comprised the study sample. Of the remaining 125 participants who were lost during the follow-up, contact could not be established with 45 (36%), 25 (20%) had died, 25 (20%) refused to participate, 21 (17%) changed their address, and 9 (7%) were not well enough to participate. The baseline characteristics of the followed-up sample are shown in the first column of Table 1. There were no significant differences in baseline characteristics between the participants and non-participants at follow-up, including the baseline blood assays of interest (data not shown, all p>0.05).

The mean (SD) change in MMSE-K score among the 607 participants over the 2.4-year follow-up was -1.3 (3.9) points. Associations between baseline characteristics and cognitive decline are displayed in the second through third columns of Table 1. Cognitive decline was significantly associated with more severe disability, higher vascular risk scores and the presence of the APOE e4 allele. There was no association between cognitive decline and vitamin intake at the final follow-up (p=0.456).

**FIGURE 1:**
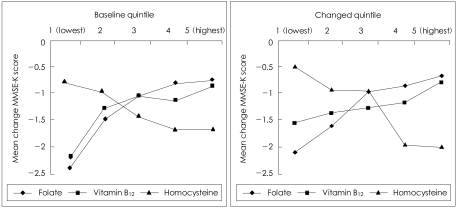
Mean changes in Korean version of Mini-Mental State Examination (MMSE-K) scores according to baseline levels of folate, vitamin B_12_ and homocysteine and changes in these levels over a 2-year follow-up period.

The two graphs in Figure 1 plot the mean change in MMSE-K score according to the baseline quintiles of folate, vitamin B_12_ and homocysteine, and the changes in the quintiles in these levels over the follow-up period. The mean changes in the MMSE-K scores across ascending quintiles of baseline folate levels were -2.4, -1.5, -1.1, -0.8 and -0.7 (F=2.728; p=0.029). The mean changes in the MMSE-K scores across ascending quintiles of baseline vitamin B_12_ levels were -2.2, -1.3, -1.1, -1.1 and -0.9 (F=1.584; p=0.177). The mean changes in the MMSE-K scores across ascending quintiles of baseline homocysteine levels were -0.8, -0.9, -1.4, -1.6 and -1.7 (F=1.113; p=0.350). The mean changes in the MMSE-K scores across ascending quintiles of change in folate levels (decrease to increase) over the follow-up period were -2.2, -1.7, -1.0, -0.9 and -0.7 (F=2.571; p=0.037). The mean changes in the MMSE-K scores across ascending quintiles of change in vitamin B_12_ levels were -1.5, -1.4, -1.4, -1.3 and -0.8 (F=0.532; p=0.712). The mean changes in the MMSE-K scores across ascending quintiles of change in homocysteine levels were -0.5, -0.9, -1.0, -2.0 and -2.1 (F=3.159; p=0.014).

The adjusted associations between these factors are displayed in Table 2. In summary, cognitive decline was associated with decreasing quintiles of folate at baseline, a relative decline in folate and an increase in homocysteine levels across the two examinations. These associations did not change after adjustment for disability, vascular risk scores, and APOE genotype.

## Discussion

In this prospective study of a community population, lower folate levels predicted cognitive decline over a 2.4-year follow-up period, but no associations were found between baseline cognitive decline and the levels of vitamin B_12_ or homocysteine. Over the follow-up period, cognitive decline occurred more frequently in subjects showing a relative decline in folate or a relative increase in homocysteine levels.

A particular strength of this study was that prospective data were obtained not only for cognitive status, but also for the exposures of interest. Furthermore, a large number of potential confounding factors were considered in the analyses. The follow-up rate was reasonable and did not differ with respect to the risk factors of interest. The principal limitations of the study were that cognitive function was assessed by the MMSE-K alone and detailed constituents of vitamin preparations were not collected. In addition, micronutrient values were only measured at two time points, which might have caused measurement errors or may not reflect dynamic changes from baseline to follow-up.

A common finding of previous cross-sectional clinical and community studies has been that lower levels of folate, and/or vitamin B_12_, and/or higher levels of homocysteine are associated with cognitive impairment.^1^-^5^ Cross-sectional analyses of the same participants from the previous study showed similar results.^8^ However, cross-sectional studies are limited in the extent to which causal relationships can be inferred since impaired cognitive states may lead to dietary changes that can influence the observed blood levels. The findings from more recent prospective studies have been inconsistent.

Folate is important in one-carbon metabolism, and methylation reactions involving folate may be important in the formation and maintenance of neuronal and glial membrane reactions.^17^ Most longitudinal studies have found associations between folate and cognitive decline,^18^-^20^ but one study did not.^7^ In our study, this association was not explained by other potential confounding factors, such as disability, vascular risk factors, and APOE genotype. The previous studies were primarily focused on homocysteine levels. Homocysteine is not only a marker of dysfunction in one-carbon metabolism but may also exert direct neurotoxic effects. The results of longitudinal studies have been inconsistent, with some finding significant associations,^6^,^18^,^20^,^21^ but others finding no association.^7^,^22^ Baseline homocysteine levels did not predict cognitive decline in the present study. Vitamin B_12_ is required for the synthesis of S-adenosylmethionine, which is the major methyl donor in many important methylation reactions in the central nervous system. However, cross-sectional associations with cognitive impairment have not been replicated in most longitudinal studies,^7^,^19^ including ours. An important limitation of most research is that circulating B_12_ is only a proxy marker of cobalamin deficiency at the cellular level. It has been suggested that holotranscobalamin may be a more accurate marker of risk.^23^ We did not have data on this factor, and therefore, negative findings with respect to B_12_ should be treated with caution.

Very few studies have examined the changes in the levels of these factors. In a recent longitudinal study, assays were carried out six years apart, and a decrease in folate and an increase in homocysteine were found to be associated with cognitive decline over the same period.^19^ In our study, cognitive decline was associated with unfavorable changes in these factors. These changes were stronger correlates than baseline levels. Although our findings may be explained by the effects of such changes on neurodegenerative processes, they more strongly suggest an effect of degenerative processes on the levels of these factors. However, it is possible that both processes occur because the effects of neurodegenerative processes (for example on dietary intake) may give rise to an adverse biochemical environment, which, in turn, induces further neurodegeneration or exacerbates its effects on cognitive function. Further research is required in order to clarify these longitudinal interrelationships. In the meantime, attention needs to be paid to the nutritional status of people with cognitive decline from the time of detection, regardless of whether this is a cause or effect of their condition.

## Figures and Tables

**TABLE 1:**
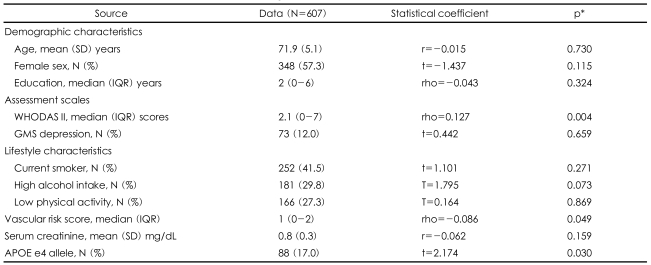
Baseline characteristics and associations with cognitive decline

^*^By t-, Pearson's correlation, or Spearman's correlation tests as appropriate. IQR: interquartile range, WHODAS II: World Health Organization Disability Assessment Schedule, GMS: Geriatric Mental State schedule, APOE: apolipoprotein E

**TABLE 2:**
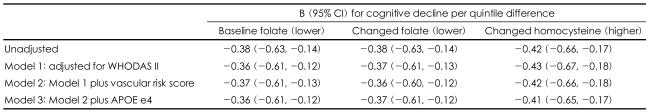
Linear regression models for the associations between cognitive decline and baseline folate levels and changes in folate/homocysteine quintiles over the 2.4-year follow-up period (N=607)

WHODAS II: World Health Organization Disability Assessment Schedule, APOE: apolipoprotein E
